# Time intervals from first symptom to treatment of cancer: a cohort study of 2,212 newly diagnosed cancer patients

**DOI:** 10.1186/1472-6963-11-284

**Published:** 2011-10-25

**Authors:** Rikke P Hansen, Peter Vedsted, Ineta Sokolowski, Jens Søndergaard, Frede Olesen

**Affiliations:** 1 Research Unit and Department of General Practice, Aarhus University, Bartholins Allé 2, DK-8000 Aarhus C, Denmark; 2 The Danish Cancer Society and the Novo Nordisk Foundation Research Centre for Cancer Diagnosis in Primary Care, Bartholins Allé 2, DK-8000 Aarhus C, Denmark; 3 Research Unit for General Practice, Aarhus University, Bartholins Allé 2, DK-8000 Aarhus C, Denmark; 4 Research Unit for General Practice, Institute of Public Health, University of Southern Denmark, J.B. Winsløws Vej 9, DK-5000 Odense C, Denmark

## Abstract

**Background:**

Delay in diagnosis of cancer may worsen prognosis. The aim of this study is to explore patient-, general practitioner (GP)- and system-related delay in the interval from first cancer symptom to diagnosis and treatment, and to analyse the extent to which delays differ by cancer type.

**Methods:**

Population-based cohort study conducted in 2004-05 in the County of Aarhus, Denmark (640,000 inhabitants). Data were collected from administrative registries and questionnaires completed by GPs on 2,212 cancer patients newly diagnosed during a 1-year period. Median delay (in days) with interquartile interval (IQI) was the main outcome measure.

**Results:**

Median total delay was 98 days (IQI 57-168). Most of the total delay stemmed from patient (median 21 days (7-56)) and system delay (median 55 days (32-93)). Median GP delay was 0 (0-2) days. Total delay was shortest among patients with ovarian (median 60 days (45-112)) and breast cancer (median 65 days (39-106)) and longest among patients with prostate (median 130 days (89-254)) and bladder cancer (median 134 days (93-181)).

**Conclusion:**

System delay accounted for a substantial part of the total delay experienced by cancer patients. This points to a need for shortening clinical pathways if possible. A long patient delay calls for research into patient awareness of cancer. For all delay components, special focus should be given to the 4^th ^quartile of patients with the longest time intervals and we need research into the quality of the diagnostic work-up process. We found large variations in delay for different types of cancer. Improvements should therefore target both the population at large and the specific needs associated with individual cancer types and their symptoms.

## Background

The time interval from first symptom to start of treatment (i.e. surgery, chemotherapy) is often labelled delay even if parts of this delay are unavoidable. Delay in cancer diagnosis is common and long delay is associated with significant mental strain and possibly a worsened prognosis [1-3]. Although the exact effect of delay on clinical outcomes remains unclear and varies between cancers, it is generally accepted that total delay should be as short as possible [4-11]. British studies attribute that long delay may account for 5-10,000 extra deaths in the UK [2,12]. Only a few studies have analyzed in detail how delay is related to patients' health-seeking behaviour, general practitioners' (GPs') clinical performance and system-related factors such as logistics, waits and administrative procedures. Moreover, previous studies have covered only one or few specific cancer types [13-18]. Cancer represents one of the most severe diseases requiring the concerted effort of the entire care system. It is therefore an excellent candidate for a study of the interplay between the elements forming part of the overall clinical pathway. Knowledge of this may enable targeted efforts to improve the organization of care. The aim of this study was to conduct a population-based cohort study examining patient-, GP- and system-related delay and the extent to which delays vary by cancer type.

## Methods

### Study design

**Figure 1:**
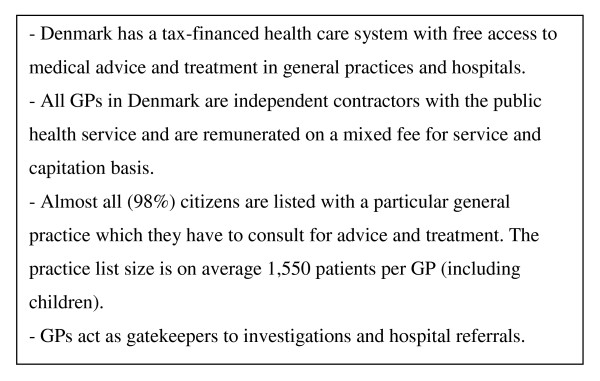
The Danish health care system.

We performed a cohort study in the Danish County of Aarhus, which has 640,000 inhabitants, approx. 3,000 new cancer cases per year and approx. 500 GPs. Denmark's publicly funded health-care system provides free access to primary care and outpatient and hospital care (Figure 1). More than 98% of the Danish citizens are registered with a GP [19,20]. GPs function as gatekeepers to the rest of the health-care system, carrying out initial diagnostic investigations including referral to initial x-ray and endoscopic procedures When indicated, they refer patients to hospitals or outpatient clinics, thus delegating further responsibility to secondary care. Danish GPs are required to keep detailed electronic records.

We included all newly diagnosed cancer patients during the 1-year period from 1 September 2004 to 31 August 2005. Patients younger than 18 years and patients with non-melanoma skin cancers were excluded. Patients were enrolled based on computerised data from the county hospital discharge registry (HDR), which for each hospital admission and outpatient visit records the patient's civil registration number (CRN), dates of admission and discharge, surgical procedure(s) performed and discharge diagnoses classified according to the international classification of diseases (ICD-10). We included all patients listed in the registry during the study period with incident cancer diagnoses, i.e. we excluded those with a recurrent cancer. Patients with previous cancer, but of another type, were regarded as incident cases. We then linked the HDR data to the county's Health Service Registry (HSR) in order to identify each patient's GP. The personal identifier used to link records to individuals across Danish registries is the CRN, which is assigned to all Danish citizens [21].

### Ethics approval

According to the Scientific Ethics Committee in the County of Aarhus, the project did not need approval by the Danish Biomedical Research Ethics Committee System. The study was approved by the Danish Data Protection Agency and the Danish National Board of Health.

### Data collection

A questionnaire was sent to the GP of each patient identified in the HDR. The GP was asked to confirm the diagnosis and to give a detailed description of the patient's diagnostic pathway using the dates reported in the medical record and the mandatory discharge letters from hospitals and specialists which report the date of first admission, the date of diagnosis and of first treatment. Furthermore, the GP provided the dates of the first patient symptoms (adherent to the actual cancer disease) as reported to the GP by the patient, the first presentation to the GP/practice, the initiation of diagnostic procedures, hospital referral, the first hospital visit, the diagnosis and the treatment start. Non-responders received a reminder after three weeks. The questionnaire was pilot-tested by the scientific staff and lay persons at the Department and Research Unit for General Practice, Aarhus and by 40 GPs from Vejle County, Denmark (purposefully selected by gender, age and practice location) [22].

### Outcome measures

**Figure 2:**
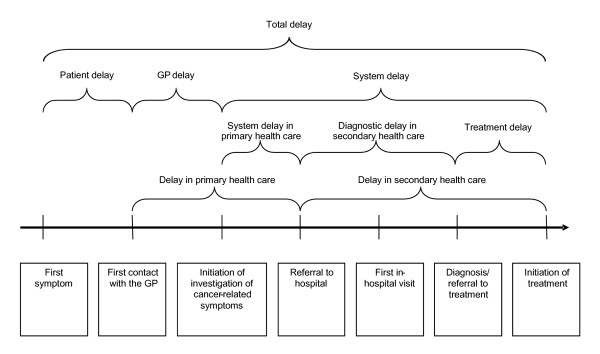
Subdivision of delay.

In accordance with most other research we used the term delay when calculating the different time intervals from first symptom to treatment start. Delay was calculated from the dates entered in the questionnaires by the GPs. Delay was divided into three delay stages. (1) Patient delay: Time from first perceived patient symptom until first presentation to the GP; (2) GP delay: Period from first presentation until initiation of an investigation of potentially cancer-related symptoms, i.e. biopsy, referral to imaging diagnostic, endoscopy or to other health-care professionals; (3) System delay: Time from the start of the GP-initiated investigation until the start of treatment. System delay was further divided into delay encountered in the primary health care sector (GP retaining responsibility for the patient) and in the secondary health care sector (the patient is no longer the responsibility of the GP) (Figure 2). System delay was defined in this way to be able to analyse to which extent cancer was seen and acted upon as an acute disease. In addition, total delay was defined as the interval from the first perceived patient symptom until treatment start.

We first computed delay for all cancer types and then for the 10 most frequent cancers found among the patients. The delay data are presented as medians and interquartile intervals (IQIs). The Wilcoxon rank-sum test was used to compare delay for those patients whose GPs were involved in the diagnostic investigation and those whose GPs were not. A P-value of 0.05 or less was used to signify statistical significance. Data were analysed using Stata software, version 11 (StataCorp, College Station, Tex).

## Results

### Descriptive data

**Figure 3:**
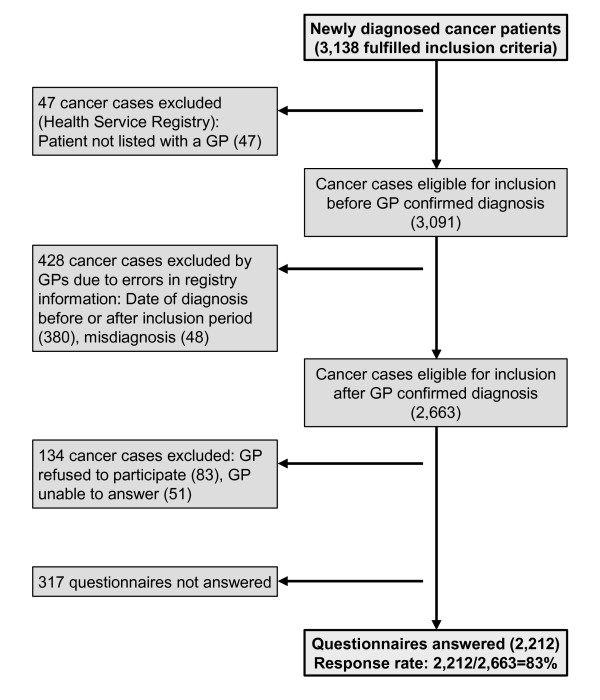
Flowchart.

A total of 2,212 questionnaires were completed. A total of 467 out of 543 physicians from 255 general practices participated in the study. On average, they filled in questionnaires on 4.7 patients (range 1-15, median 4). The physician response rate was 83% (Figure 3). No significant differences were found between the participating and non-participating physicians concerning gender, practice organization and years since graduation. GPs were involved in the diagnostic pathway in 1,892 (86%) of the 2,212 cancer cases. For the remaining 320 cancer cases, we could only calculate system delay in the secondary health-care sector because they were admitted directly to the hospital by out-of-hours or accident and emergency services (A&E), often for acute-onset symptoms. Table 1 shows the distribution of the 10 most frequent cancers diagnosed in Denmark [23] compared with the cancer distribution by type in our study.

**Table 1:** The number and the distribution of the 10 most frequent cancers diagnosed in Denmark and the cancer distribution by type in the study

Cancers in DK (N = 33501 in 2003)*	Cancers in this study (N = 2212)
1. Breast cancer (4027 - 12.0%)	1. Lung cancer (328 - 14.8%)
2. Colorectal cancer (3576 - 10.7%)	2. Breast cancer (311 - 14.1%)
3. Lung cancer (3500 - 10.4%)	3. Colorectal cancer (291 - 13.2%)
4. Prostate cancer (2378 - 7.1%)	4. Prostate cancer (214 - 9.7%)
5. Bladder cancer (1710 - 5.1%)	5. Melanoma (131 - 5.9%)
6. Melanoma (1222 - 3.6%)	6. Bladder cancer (80 - 3.6%)
7. CNS cancer (898 - 2.7%)	7. Non-Hodgkin's lymphoma (65 - 2.9%)
8. Non-Hodgkin's lymphoma (828 - 2.5%)	8. Pancreas cancer (63 - 2.8%)
9. Pancreas cancer (700 - 2.1%)	9. Ovarian cancer (59 - 2.7%)
10. Leukaemia (694 - 2.1%)	10. Corpus uteri cancer (47 - 2.1%)

* Including cancers in patients younger than 18 years

### Delay data

Table 2 shows the different delay stages for all cancers and for the 10 most frequent cancers diagnosed among study patients. Overall, among patients with complete questionnaire data the median total delay was 98 days (IQI 57 to 168), the median patient delay was 21 days (IQI 7 to 56), the median GP delay was 0 days (IQI 0 to 2) and the median system delay was 55 days (IQI 32 to 93). The period from referral to treatment (system delay in secondary health care) accounted for most of the system delay (median 46 days, IQI 26 to 78), while system delay in primary health care was minimal (median 0 days, IQI 0 to 12). Patients who saw their GPs prior to diagnosis experienced a statistically significantly (Z = 3.257, P = 0.001) longer system delay in secondary health care (median 46 days, IQI 26 to 78) than those who were admitted directly to the hospital (median 37 days, IQI 17 to 63).

**Table 2:** Delay (in days) for all and the 10 most frequent cancers in the study when the GP was involved in the diagnostic investigation process

										System delay																	
													
	Total delay			Patient delay			GP delay									System delay in secondary health care									System delay in secondary health care (GP not involved)		
																			
										Total system delay			System delay in primary health care			System delay in secondary health care			Diagnostic delay in secondary health care			Treatment delay					
**Cancer type (Cancer cases, GP involved/Cancer cases, GP not involved)**	**N**	**Median**	IQI*	**N**	**Median**	**IQI**	**N**	**Median**	**IQI**	**N**	**Median**	**IQI**	**N**	**Median**	**IQI**	**N**	**Median**	**IQI**	**N**	**Median**	**IQI**	**N**	**Median**	**IQI**	**N**	**Median**	**IQI**

All cancers (1892/320)	936	98	57-168	1237	21	7-56	1877	0	0-2	1422	55	32-93	1874	0	0-12	1412	46	26-78	1823	29	14-57	1403	14	0-28	174	37	17-63

Breast cancer (291/20)	159	65	39-106	168	14	0-56	289	0	0-0	269	40	25-59	287	0	0-0	268	37	22-51	281	21	13-36	266	12	4-20	10	29	23-38

Colorectal cancer (254/37)	159	109	65-194	187	28	14-56	254	0	0-6	212	56	34-87	254	0	0-10	216	48	28-71	251	30	15-50	215	14	0-28	24	15	3-30

Lung cancer (253/75)	128	108	82-167	182	28	7-56	251	0	0-9	182	69	47-96	250	7	0-18	181	55	36-79	246	27	14-46	182	23	8-36	40	51	27-76

Prostate cancer (190/24)	73	130	89-254	110	28	0-112	186	0	0-6	117	102	55-151	187	6	0-18	115	75	44-135	183	80	44-110	114	9	0-29	16	83	48-139

Melanoma (122/9)	38	114	79-279	40	70	28-196	122	0	0-0	114	39	22-65	122	0	0-15	112	27	15-54	117	20	0-42	110	13	0-23	1	49	

Bladder cancer (73/7)	32	134	93-181	52	14	0-28	72	0	0-10	43	91	52-119	71	2	0-19	42	80	45-113	68	62	35-87	43	0	0-20	4	51	22-75

Non-Hodgkin's lymphoma (54/11)	29	78	58-118	43	21	7-42	53	0	0-3	34	60	44-86	54	2	0-14	34	55	39-73	51	36	14-68	34	12	5-29	4	38	33-51

Pancreas cancer (54/9)	21	87	49-153	40	14	7-42	53	0	0-6	28	63	27-117	54	0	0-15	28	59	25-91	51	27	11-68	27	14	0-41	5	15	10-50

Ovarian cancer (47/12)	32	60	45-112	38	21	0-35	47	0	0-2	40	30	21-49	47	0	0-8	40	26	20-43	45	21	12-31	39	1	0-21	9	40	20-62

Corpus uteri cancer (41/6)	35	99	51-22	35	21	0-140	41	0	0-0	41	64	41-106	41	0	0-1	41	60	32-101	39	38	23-79	39	7	0-14	3	59	28-77

Others (513/110)	233	96	56-178	342	21	7-56	509	0	0-6	338	63	35-110	507	0	0-11	335	52	29-93	491	27	12-55	334	17	0-34	58	32	13-62

N in each column is the number of answers with complete data. Last column shows system delay in secondary health care when the GP was not involved in the diagnostic investigation process

*IQI = interquartile interval

**Figure 4:**
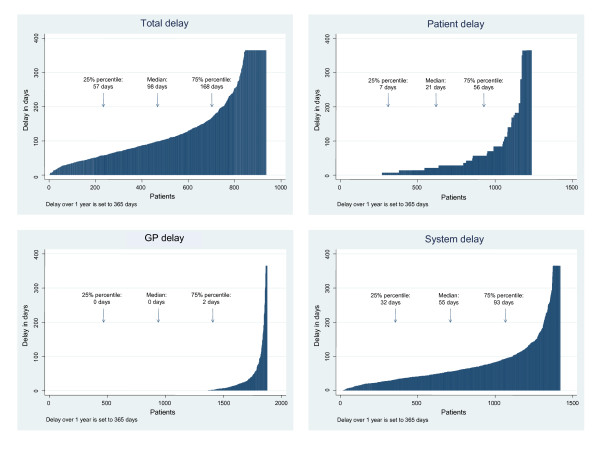
Total, patient, GP and system delay. Delay (in days) for each patient is shown in the figures.

The shortest total delay was seen among patients with ovarian cancer (median 60 days, IQI 45 to 112); the second shortest delay was seen among patients with breast cancer (median 65 days, IQI 39 to 106). The longest total delay was found among patients with prostate (median 130, IQI 89 to 254) and bladder cancer (median 134, IQI 93 to 181) (Table 2). As shown in Figure 4, the 4^th ^quartile of patients experienced long patient, GP, system and total delay. Non-response analysis revealed no major differences between participating and non-participating physicians in terms of their cancer patients' age, gender or distribution of cancer diagnoses.

## Discussion

We identified a median total delay of 98 days and a total delay exceeding 168 days among ¼ of the patients. Patient and system delay accounted for most of the total delay. Even if the median GP delay was 0 days, a considerable proportion of the patients experienced a long GP-related delay. We found much variation in delay by cancer type. As expected, patients who saw their GPs prior to diagnosis experienced longer delays than those who accessed specialist care directly.

In this paper we alternate between the terms "delay" and "time interval". Strictly speaking, the use of the term "delay" is often inappropriate as part of the time interval is unavoidable, and therefore the term "time interval" is often more correct. As delay is still widely used in the literature we have for comparable reasons continued to use this term, but future investigations might more consequently change the term to time interval.

The study covered the entire population above 17 years with newly diagnosed cancer in a large catchment area counting more than 600,000 inhabitants. The study is thus based on data on the total health-care performance in relation to cancer within a region during a 1-year period.

We reduced selection bias by using registry information to identify potential study participants independently of the participating GPs and hospital physicians. The excellent response rate (83%) limits a possible selection bias; but, still, non-responding GPs may have seen patients with special diagnostic pathways, although non-response analysis revealed no major differences between participating and non-participating physicians in terms of their cancer patients' age, gender or distribution of cancer diagnoses. The description of socioeconomic patient characteristics associated with delay was not within the scope of this article, but this issue is covered in another article [24].

We further assured that only eligible patients were included by requesting that their GPs confirmed their diagnoses. Given the uniform organisation of health care throughout Denmark, we consider our results representative of the country as a whole. We cannot determine whether our findings are representative of other cultural settings or health-care systems in other counties. We recognize that the sample sizes of some of the delay calculations (especially total delay) for 6 of the 10 cancer types in the study (Table 2) are small (due to missing data and a small number of these cancers in Denmark). This may have affected the IQIs and evidently the generalisability of the results.

Minimisation of recall bias is a key prerequisite for the validity of our findings. We therefore encouraged the GPs to consult their electronic patient files including the discharge letters when filling in the questionnaires. Still, lack of complete information in some questionnaires may have introduced information bias. We anticipate that this bias tends to underestimate the reported delay, which may especially be the case for GP-reported delay. In another paper we have shown that it is difficult to define the time of symptom onset [25]. However, we do not yet know whether the patients' or the GPs' estimates are the most correct ones.

### Comparison of findings with previous literature

Seen from a health services planning and a research and population perspective, it is relevant to include all cancers to be able to formulate a hypothesis about the need to focus on patient awareness of symptoms and access to diagnostic work-up of serious disease. However, our data on the specific cancer types also emphasise the need for detailed analyses of each specific cancer. We also need more research on the influence of symptom presentation in relation to delay [26]. A previous survey from the same region by Bjerager [13] showed that most delay in diagnosing lung cancer was attributable to system delay, which is consistent with our findings.

Our total delay findings accord with those of Allgar and Neal [17] who investigated patient-reported delay for six cancer types in a large population of UK cancer patients. However, in contrast to our results, they found that the main problem was patient and primary care delay. This discrepancy may be attributed to differences in the English and Danish health care systems in terms of culture, organisation and capacity, and to differences in study design and definitions of delay stages. Our broad definition of system delay encompassed not only time to diagnosis, but the time to start of treatment, as we expected a significant delay between diagnosis and treatment initiation. We need an internationally agreed definition on delay stages (Figure 2); a task that has been initiated in the UK National Initiative on Early Diagnosis of Cancer (NAEDI) and in the Cancer and Primary Care Research International Network (CA-PRI) [2,27].

### Implications of the study

Cancer is a serious disease. The diagnostic evaluation is complex and often conducted in a sequential process, which may explain the long system delay. However, another explanation may be that Danish cancer patients have to wait longer for basic diagnostic investigations. Thus, another main finding of the present study was the long patient delay. We lack international knowledge about variation in patient awareness of symptoms and differences in delay and about the interaction between the health care structure and patient delay [28-30].

A recent paper showed that delay in diagnosis may have severe prognostic consequences and may partly explain the bad cancer outcome results in countries like the UK and Denmark [12]. Recent years have therefore seen the introduction of different initiatives to reduce system delay in the UK. However, a fast-track approach based on a GP's suspicion of cancer may not necessarily be effective as only some 50% of patients present to their GP with alarm symptoms [26,31]. The effect of seeing cancer as an acute disease and thereby circumventing administrative and capacity-driven bottlenecks in the pathway should be investigated. The fast-track approach was adopted in Denmark in 2008 [1,27], but it is too early to evaluate if this initiative has reduced system-related delay.

Further research is required to test whether improved access to diagnostic investigations and intra- and inter-sectoral cooperation within the health care sector will have an effect on the diagnosis of cancer.

Further analysis of the long delays characterising the 4^th ^quartile of patients requires clarification of the specific patient, GP and system characteristics that prolong delay. Our investigations invite the conclusion that a more precise research focus on early diagnosis of cancer is, indeed, warranted [2].

## Conclusions

This study showed that patient and system delay account for most of the delay in cancer diagnosis. For all components of delay, special focus should be given to the 4^th ^quartile of the patients. Cancer prognosis may be improved if we focus on early diagnosis and improved clinical pathways.

## Competing interests

The authors declare that they have no competing interests.

## Authors' contributions

FO conceived the study. The study was conducted by RPH in consultation with all the co-authors, IS performed the statistical analyses in consultation with the other authors. RPH drafted the manuscript and all authors contributed to critically revising the paper. Finally, all authors read and approved the submitted manuscript.

## Funding

The study was funded by grants from the Danish Agency for Science Technology and Innovation -the Danish Medical Research Council (22-03-0208), the Pharmaceutical Foundation of 1991 (139-2003), the Aarhus County Research Fund for the Clinical Development and Research in General Practice and Across the Primary and Secondary Health Care Sectors (4-01-2-2-02/4-01-2-5-00/4-01-3-04), and the Regional Clinical Research Unit of the Danish Cancer Society - Region North (KFE-AA-289-03). The funding sources had no involvement in the research process.

## Pre-publication history

The pre-publication history for this paper can be accessed here:

http://www.biomedcentral.com/1472-6963/11/284/prepub
